# An innovative method for occluding the urethral meatus and accessing urethra strictures in retrograde urethrography in males

**DOI:** 10.1186/s12894-023-01328-0

**Published:** 2023-10-06

**Authors:** Wei Li, Libo Man, Guanglin Huang

**Affiliations:** Department of Urology Surgery, Beijing Jishuitan Hospital, Capital Medical University, Beijing, 100069 People’s Republic of China

**Keywords:** Urethral stricture, Urethrography, Sponge plug, Urethra external meatus

## Abstract

**Objectives:**

To observe and evaluate the effectiveness and safety of using a sponge plug method to occlude the urethral meatus during retrograde urethrography (RUG) for accessing male urethral strictures.

**Methods:**

40 male patients with a mean age of 51.4 years and a history of urethral injury were primarily diagnosed with urethral stricture using a urethrocystoscope. RUG was performed using a ureteral catheter with a sponge plug inserted into the external meatus. Iodixanol, a contrast medium, was injected into the urethra or bladder for performing RUG and voiding cystourethrography (VCUG). The patients were positioned obliquely to obtain urethrograms.

**Results:**

All X-ray radiologic procedures for performing urethrography were successful without any overflow of contrast liquid observed. In all cases, the sponge plugs became visible in the resulting images. The external meatuses were directly visualized in all cases on the obtained images, allowing identification of the number, location, and length of strictures as well as coexistent pathologies such as fistulas. In one case, the plug slipped off the meatus immediately after completing the procedure. The pain Visual Analogue Scale (VAS) was 0 to 2, mean 0.35. No instances of complication or adverse reactions was observed.

**Conclusions:**

The sponge plug effectively occludes the external urethral meatus for retrograde urethrography, enabling visualization of the actual caliber of the entire urethra, including the strictures and external meatus, by filling it with contrast liquid. This technique is safe and well-tolerated by patients.

## Background

X-ray urethrography remains the essential procedure of assessing the urethra stricture disease [1], which includes retrograde urethrography (RUG) and voiding cystourethrography (VCUG) [2]. For fulling urethral lumen with contrast liquid to visualize actual urethral caliber, the external meatus of urethra should be blocked off to avoid contrast running out of the meatus [3, 4]. Conventional options include Foley’s catheter [2], penis clamp [3, 4], meatus adapter [5]. This study introduces an innovative device for occluding the meatus, and its efficacy and safety are assessed.

## Methods

### Patients

After obtaining approval from our institutional ethics committee, this study enrolled 40 male patients with a history of urethral injury, including pelvic fracture urethral injury (PFUI), straddle urethral injury, or iatrogenic injury resulting from transurethral operations. These patients were scheduled to undergo urethral surgery at Beijing Jishuitan Hospital between January and December 2022. In order to exclude other lower urinary tract diseases, all patients had initially been diagnosed with urethral stricture via cystourethroscopy and were already equipped with suprapubic catheters due to urinary retention. Furthermore, RUG and VCUG were required for all cases to access both the proximal and distal segments of the stricture. All methods were carried out in accordance with relevant guidelines and regulations. In all cases, informed consent was obtained after providing a detailed explanation of the innovative urethrography procedure.

### Procedures

The urologist performed all urethra manipulations following these steps:

The penis was disinfected with iodophor after retracting the foreskin.

A 7-Fr ureteral catheter (ST-250,700, UROVISION, Germany) was passed through a sponge plug (1100, 3 M, USA) along the vertical axis. Both the catheter and plug were sterilized by ethylene oxide.

**Fig. 1:**
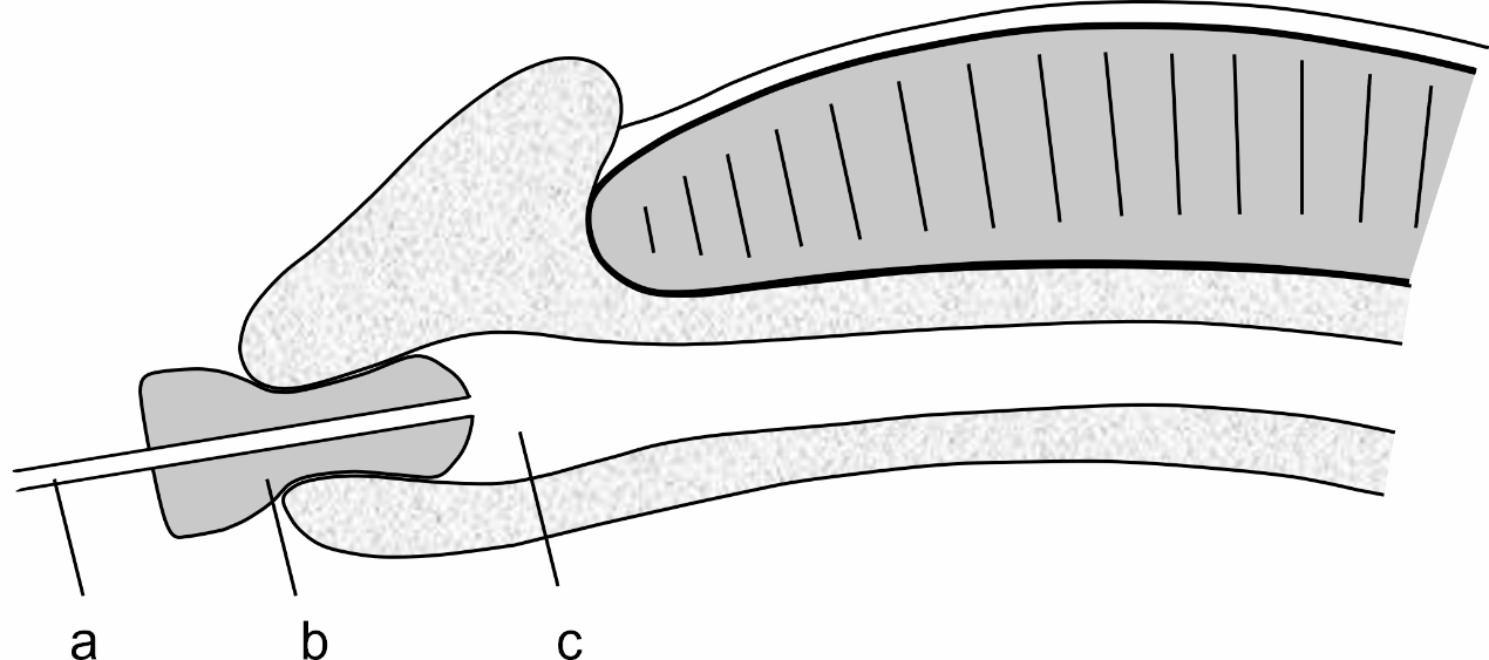
The sponge plug was inserted into the urethral meatus (longitudinal section). (**a**) The ureteral catheter; (**b**) the sponge plug; (**c**) the lumen of the urethra

The urologist compresses the sponge plug to make it thin and then inserts it into the anterior urethra, 1.5 cm deep from the meatus orifice. After about 10 s, the compressed sponge plug swells and returns to its original shape until it reaches an appropriate size for the meatus lumen and fossa navicularis. (Fig. 1).

The patient should be in the oblique supine position (30 to 45 degrees) while maintaining the penis stretched to maximize visualization of the anterior urethra [6, 7]. The Iodixanol-based contrast medium (160 mg I/ml) is slowly injected through the suprapubic catheter to fill the bladder. The contrast medium needs to be injected from the meatus through the catheter with the sponge plug to fill the anterior urethra. The sponge plug should be left in place for several seconds to allow absorption of the contrast medium. Meanwhile, its effect on outflow should be observed beside the patient. If the obstruction is not complete, then the contrast injected in RUG will be lost in the bladder. However, this backflow is not complete and the contrast medium will fill the lumen during VCUG. In our practical observations, the penis could be straight spontaneously after injection in the urethra lumen, and the penis was put on the leg to keep urethra unfolding. Subsequently, the urologist can leave the room and observe the monitor screen to avoid exposure to radiation. Under X-ray urethrography guidance, the patient is instructed to void until both the bladder neck and proximal urethra are filled, allowing for a comprehensive evaluation of the entire segment of urethral stricture and any associated pathologies. Sometimes, a few patients are unable to void for VCUG, we prefer teaching them how to relax the sphincter and encourage them to void.

Multiple radiographic images are acquired using the X-ray machine. (Sonialvision Safire II / 4124A3326005, SHIMADZU, Japan).

Once the X-ray radiographic examination is completed, the catheter and sponge plug can be easily removed, and they are intended for single use only.

### Data collection

The data were acquired and collected by professional urologists throughout the entire process to ensure the utmost quality of the data.

It was observed whether the stricture segments were clearly shown as filling defects, and both the urethra and sponge plug became visible under X-ray.

The characteristics of stricture segments (location, number, length, associated pathology) were recorded from both X-ray urethrography via PACS software and the urethroplasty procedure.

The level of pain was measured using the Visual Analog Scale (VAS), which ranges from 0 to 10.

### Statistical analysis

Continuous data is presented as the mean ± SD or median with ranges, while categorical data is expressed as frequencies and percentages. The differences in measurement data were compared using a paired t-test, where *p* < 0.05 was considered statistically significant. Statistical analyses were performed using SPSS ver.19.0 (SPSS Inc., Chicago, IL, USA).

## Results

This study enrolled 40 male patients with a mean age of 51.4 years (age range: 23–72 years). All the X-ray radiologic urethrography procedures were performed successfully. Some characteristics of strictures are presented in the Table 1. The location and number of strictures were consistent with the surgical procedure. A comparison was conducted between the length of strictures observed in urethrography (15.53 ± 2.09 mm) and during the operation (15.20 ± 1.99), *p* > 0.05.

**Table 1:** Characteristic of cases

Characteristic		n	%
Etiology			
	PFUI	15	37.5
	straddle	11	27.5
	iatrogenic	14	35
Location			
	internal orifice	1	2.5
	prostatic	4	10
	membranous	19	47.5
	bulbar	13	32.5
	penile	3	7.5
Number			
	single	39	97.5
	multiple	1	2.5
VAS			
	0	29	72.5
	1	8	20
	2	3	7.5
Adverse reaction		0	0
Overflow		0	0
Slip off		1	2.5

**Fig. 2:**
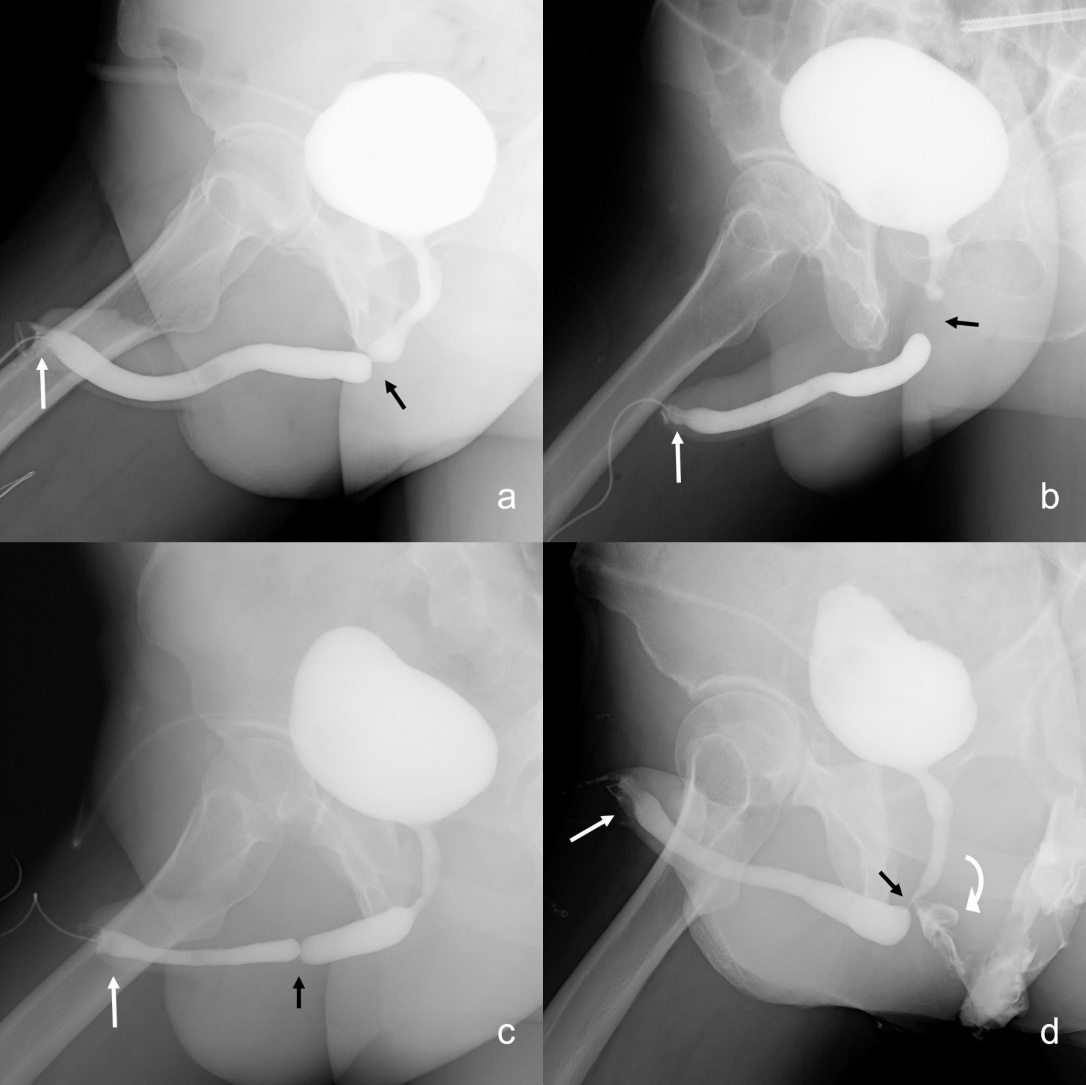
The urethrograms were obtained through RUG and VCUG. The strictured segments were directly visualized due to the opacified urethral lumen at both ends of the strictures (black arrow). The opacified plugs clearly displayed the structures of the urethral meatus (white arrow). Each case had a specific history and location: (**a**) straddle injury involving the bulbar urethra, (**b**) pelvic fracture affecting the membranous urethra, (**c**) iatrogenic injury to the penile urethra. (**d**) exhibited a urethral fistula indicated by the curved arrow

In all cases, the sponge plugs appeared opaque in the urethrograms, which directly visualized the actual lumen of the meatus. In one case, the plug slipped off the meatus immediately after completing the procedure. The obtained urethrograms showed urethral strictures in all cases (Fig. 2a and c), and coexisting urethral pathology, such as fistula, could also be identified (Fig. 2d).

The pain Visual Analogue Scale (VAS) ranged from 0 to 2, mean 0.35, as shown in Table 1. No instances of overflow, complications, or adverse reactions were observed.

## Discussion

X-ray urethrography remains the essential procedure for assessing urethral stricture disease [1]. Simultaneous RUG and VCUG allow precise assessment to define the location, length, number, and severity of strictures. These factors will guide management decisions, especially for obliterative strictures where it is important to determine the full extent [2, 5, 8–10]. In this study, we advocate performing both RUG and VCUG in the same sitting. Furthermore, these key factors directly affect the choice of surgical methods and recurrence rate [2, 8, 11, 12] .

However, the single voiding method may not demonstrate certain abnormalities of the male anterior urethra because the normal anterior urethra is not fully distended [13]. To fully visualize the actual urethral caliber by filling the urethral lumen with contrast liquid, retrograde injection of contrast medium should be performed and the external meatus of the urethra should be blocked off to prevent contrast from running out [3, 4, 13].

Bach et al. suggested that the operating urologist may be better off performing and interpreting the urethrogram, as this would lead to the most accurate identification of strictures and description of stricture length [14]. In this study, the urologist successfully performed urethrography procedures in all cases and obtained comprehensive key characteristics of strictures. The contrast medium was retrogradely injected through a sponge plug that occluded the urethral orifice to prevent reflux.

The amount of contrast medium is controlled during the procedures, as the approximate volume of the anterior urethra is 20–30 cc [6, 10]. This volume should serve as a guide to ensure appropriate luminal distension. Under distension of the urethral lumen can lead to underestimation of the true stricture length [8], while excessive pressure may cause the plug to slip off. Additionally, lidocaine gel was not utilized in this study to enhance the success rate [15], as it could potentially cause the plug to slip off or result in contrast medium leakage [7], and its benefit for patient comfort is questionable [10].

Several reports [6, 8, 13, 15–17] have mentioned the use of a Foley catheter, in which the balloon is gently inflated just proximal to the fossa navicularis to block the urethra. This method is attractive because it avoids contrast leakage around the genitalia and also allows the investigator to keep his or her hands well clear of the field of radiation [13]. However, Ayoob et al. have reported that proper positioning of the catheter in the urethra is crucial because placing it too far inside can obscure a distal anterior urethral stricture [8]. Ramanathan et al. prefer using fluid to inflate the balloon because it is more uncompressible than air, but accidental withdrawal of the balloon can still occur during the study [6]. Excessive or overly rapid balloon distention can cause mucosal laceration, leading to intense and long-lasting pain as well as urethral bleeding. In some cases, the balloons were gradually inflated until the patients experienced pain, resulting in inevitable discomfort [15–17].

Another frequently used method is the clamp system, which tightly bands the balanoprepucial sulcus with a padded ring [13, 18]. The 2–4 cm of the distal urethra (inside the glans) is not visualized in the retrograde study [3]. Ayoob et al. reported that cine imaging or spot images at 2–4 frames/sec could be used to visualize the far anterior urethra as the catheter is removed and contrast empties [8]. However, this was not the actual lumen because the anterior urethra was not fully distended [13].

In this study, we observed several characteristics of the sponge plug. Firstly, it plugs the urethral meatus to prevent overflow of contrast liquid and maintain fluid pressure in the urethral lumen. Secondly, it can be easily pinched down and inserted into the meatus of the urethra, gradually expanding and adapting to its shape. This adaptation is not forceful or tough [5], but rather gentle, allowing the sponge to naturally conform to the shape of the orifice. Thirdly, the sponge absorbs contrast material, making the external urethral meatus visible in a urethrogram. Lastly, due to its soft and gentle insertion method, 72.5% of patients reported no pain with a VAS score of 0; while 27.5% experienced slight discomfort. However, despite these characteristics potentially being superior to other established methods, further prospective controlled trials are necessary to substantiate their efficacy.

However, the sponge plug is still ineffective for patients suffering from urethral external meatus stricture, especially lichen sclerosis. The narrow orifice requires a slimmer adapter [5], and there is not enough space for compression and expansion of the sponge. Urologists cannot accurately observe the extent of spongiofibrosis through X-ray urethrography using sponge plugs or other conventional methods. However, sonourethrography, computed tomography urethrography, and magnetic resonance urethrography may have advantages in this regard [1, 3].

## Conclusion

The sponge plug effectively occludes the external urethral meatus for retrograde urethrography, enabling visualization of the actual caliber of the entire urethra, including the strictures the external meatus, by filling it with contrast liquid. This technique is safe and well-tolerated by patients.

## Data Availability

The datasets used and/or analyzed during the current study are available from the corresponding author on reasonable request.
